# Promising Roles of Alternative Medicine and Plant-Based Nanotechnology as Remedies for Urinary Tract Infections

**DOI:** 10.3390/molecules25235593

**Published:** 2020-11-28

**Authors:** Harish Chandra, Chanchal Singh, Pragati Kumari, Saurabh Yadav, Abhay P. Mishra, Aleksey Laishevtcev, Ciprian Brisc, Mihaela Cristina Brisc, Mihai Alexandru Munteanu, Simona Bungau

**Affiliations:** 1 Department of Botany and Microbiology, Gurukula Kangri (Deemed to be University), Haridwar 249404, India; hreesh5@gmail.com; 2 Department of Microbiology, Faculty of Science and Technology, Mewar University, Chittorgarh 312901, India; 25chanchal@gmail.com; 3 S-02, Scientist Hostel, Chauras Campus, Srinagar Garhwal, Uttarakhand 246174, India; pragati27@gmail.com; 4 Department of Biotechnology, H.N.B. Garhwal University (A Central University), Srinagar (Garhwal) 246174, Uttarakhand, India; 5 Adarsh Vijendra Institute of Pharmaceutical Sciences, Shobhit University, Gangoh 247341, India; 6 Federal Research Center, Russian Scientific Research Institute of Experimental Veterinary Medicine Named after K. I. Skryabin and Y. R. Kovalenko of the Russian Academy of Sciences, 109428 Moscow, Russia; a-laishevtsev@bk.ru; 7 Laboratory of Biocontrol and Antimicrobial Resistance, Orel State University, Named after I. S. Turgenev, 302026 Orel, Russia; 8 Department of Medical Disciplines, Faculty of Medicine and Pharmacy, University of Oradea, 410073 Oradea, Romania; brisciprian@gmail.com (C.B.); briscristina@yahoo.com (M.C.B.); mihaimunteanual@yahoo.com (M.A.M.); 9 Department of Pharmacy, Faculty of Medicine and Pharmacy, University of Oradea, 410028 Oradea, Romania; sbungau@uoradea.ro

**Keywords:** UTI, *E. coli*, alternative therapy, traditional medicine, homeopathic, Ayurvedic, Unani, nanotechnology, nanoparticles

## Abstract

Urinary tract infections (UTIs) are considered to be the most common infections worldwide, having an incidence rate of 40−60% in women. Moreover, the prevalence of this disorder in adult women is 30 times more than in men. UTIs are usually found in many hospitals and clinical practice; as disorders, they are complicated and uncomplicated; in uncomplicated cases, there is no structural or functional abnormality in the urogenital tract. However, obstruction, retention of urine flow and use of catheters increase the complexity. There are several bacteria (e.g., *E. coli*, *Klebsiella pneumoniae*, *Proteus vulgaris*, etc.) successfully residing in the tract. The diagnosis must not only be accurate but rapid, so early detection is an important step in the control of UTIs caused by uropathogens. The treatment of UTIs includes appropriate antimicrobial therapy to control the infection and kill the causal microbes inside the body. A long-time usage of antibiotics has resulted in multidrug resistance causing an impediment in treatment. Thus, alternative, combinatorial medication approaches have given some hope. Available treatments considered Homeopathic, Ayurvedic, Unani, and other herbal-based drugs. There are new upcoming roles of nanoparticles in combating UTIs which needs further validation. The role of medicinal plant-based nanotechnology approaches has shown promising results. Therefore, there must be active research in phyto-based therapies of UTIs, such as Ayurvedic Biology.

## 1. Introduction

The urinary tract of human beings is composed of the kidney, ureter, urethra, urinary bladder, and genital organs (male and female). The average urine excreted by human beings is 1.4 L per day (normal range: 0.6–2.6 L/day) [1]. The normal biochemical characteristics of urine have pH = 5.8; pale yellow color to deep amber; and absence of bilirubin, RBC, protein, and pus cells. If the biochemical characteristic of urine gets altered or changed such as urine frequency, kidney inflammation then the ureter, urethra, urinary bladder, and genital organs indicate such issues related to these organs. However, the most probable reason for the above mentioned symptomatic conditions may be the result of urinary tract infections (UTIs) caused by several bacteria viz. *Escherichia coli, Klebsiella* spp, *Proteus* spp., *Pseudomonas* spp., *Enterococcus faecalis, Staphylococcus aureus, Citrobacter* spp., *Morganella* sp., *Providencia* sp., *Serratia* sp., *Mycoplasma* sp., etc. [2]. Out of these microorganisms most frequently encountered bacterial species is *E. coli* followed by *Klebsiella* spp. Human beings, particularly females, encounter UTIs due to the anatomy of the urinary tract or reproductive organ (i.e., the female urethra is short, so there is the possibility of getting access to urinary bladder more frequently, another reason being the urethral opening near sources of bacteria from the anus) [3].

Other susceptible adults include the elderly, with these patients needing urethral catheters. The low water intake/day can moreover be one of the reasons for UTI, as the flushing action of water from the urinary tract is low. The person who takes adequate water during the daytime has less probability of getting UTI due to continuous flushing or cleaning of urinary as well as reproductive organs [4,5,6,7].

The occurrence of UTIs in the female is most common as compared to male in India, as well as other developed and underdeveloped countries. The incidences of UTIs were also reported from Uganda (East Africa), Kenya, United States, United Kingdom, etc. [8,9,10]. The possible reasons for UTIs can be unprotected sex, reduced water intake, and use of infected sanitary clothes (commonly used in small villages). Common symptoms of UTIs include severe back pain; inflammation or burning sensation while urinating; cloudy, dark, bloody, or bad-smelling urine; and fever or chill. If the infections reach the kidney, then it causes pyelonephritis (inflammation of the kidney) [3,11,12]. The modern medicine such as broad-spectrum antibiotics are prescribed for the control of uropathogens. However, due to the emergence of drug resistance in bacteria, there is a need for novel antimicrobial molecules. A large proportion of the Earth’s surface is covered by marine environment, so exploration of bioactive compounds from marine organisms may prove beneficial. The secondary metabolite or the potential bioactive compounds of endophytic actinomycetes *Nocardiopsis* sp. GRG1 (KT235640) from marine brown algae, possesses antibacterial activity against uropathogens [13].

The tunicate (Ascidians, sea squirts)-associated bacteria isolated from marine environments and antimicrobial activity of these isolated bacteria were tested against pathogenic bacteria and have shown promising results [14,15].

Based on the published literature data in the most well-known databases (MDPI, PubMed, Frontiers, Scopus, etc.), this review paper focuses on the promising role of alternative medicine and nanotechnology/nanoparticles as a remedy and possible alternative treatment in UTIs. Moreover, the aim of this study was also to highlighting new or less known aspects regarding both traditional and modern medicine related to their capacity to treat and/or cure this type of infection.

It is not confirmed that men are less prone to the UTI, but it was reported by several studies across the world that women are more prone to UTIs than the male because the woman undergoes different hormonal and physical changes [16]. The normal microflora of the vagina changes according to the stage of sexual development (i.e., before/in/after puberty). The chances of getting a maximum infection are before and after puberty, due to the lack of normal microflora *Lactobacillus* spp.; another reason is sexual activeness and multiple partners. It was estimated that 40% of women experienced UTI in their lifetime, and recurrent UTI has been reported in 40% of women [17].

The UTI prevalence in different age groups and sex of South Indian peoples was studied, and it was found that UTI was common in women who are in the puberty stage and post-menopausal stage. It was also revealed that the age group 21–30 years of a married woman are more susceptible to UTI. In the case of a male who is older than 55 years, having diabetes and prostrate are more prone to UTI. Kant et al. observed 1253 pregnant women and it was found that 33% of pregnant women encountered a UTI [18].

## 2. Diagnosis of UTI

The early and accurate diagnosis of UTIs is an important step in the treatment of the disease caused by uropathogens. The treatment of UTIs includes appropriate antimicrobial therapy to control infection and kill the causal organism inside the body. Different diagnosis methods are available for the diagnosis of UTI

### 2.1. Conventional Method of Detecting UTI

#### 2.1.1. Urine Examination

In this method, morning midstream urine is collected in a universal sterile container and the microbiological investigation is performed, with the presence of motile bacteria, RBC, and the number of pus cells being determined. The presence of more than one bacterium per high power field is indicative of possible UTI [19,20]. The other known method is the urine culture and it can be done on selective and differential media (i.e., MacConkey Agar and Cysteine Lactose Electrolyte-Deficient (CLED) agar). After streaking plates on selective media are kept at 37 °C for 18–24 h, the plate is observed regarding the numbers and the characteristics of colonies [20,21].

#### 2.1.2. Dip Slide Method

This method is a modified or rapid version of the culture method. In this method, there is no need to prepare MacConkey or CLED medium in a Petri plate. The dip slide is a readymade preparation available in the market or supplied by different manufacturers such as HiMedia, Oxoid etc. In this dip slide containing double-sided or triple-sided coated differential or selective media for detection of infection causing bacteria such CLED, MacConkey, and other media.

#### 2.1.3. Nitrite Test to Detect Bacteriuria

The nitrite test is used to detect the nitrate reductase enzyme which is present in most of the uropathogens and can be detected by using this exam. As compared to other methods available, its sensitivity is less but have significant specificity. The method was developed by the Griess in 1879; he used sulphanilic acid and α-naphthylamine in dilute acid for nitrite detection. During this procedure, the urine sample collected from the patient is subjected to the addition of 2 mL of Griess reagent (representing the mixture of sulphanilic acid and α-naphthylamine); appearance of red color indicates a UTI [22].

#### 2.1.4. Leukocyte Esterase (LE) Test for Detection of Pyuria

This test allows leucocyte (or white blood cell) detection, which releases the esterase enzyme due to inflammation of ureter, kidney, urethra, and prostate (being indicative of UTI or pyuria) [23]. The principle of LE test is that the esterase released from activated neutrophil cells reacts with indoxyl carbonic acid ester; indoxyl is released by the esterase and reacts with diazonium salt and is oxidized resulting in the formation of purple color. The formation of purple color indicates the presence of leucocyte esterase. The sensitivity of LET was reported to have 70% [24]. These above tests have been represented in Table 1.

## 3. Modern Approach in the Detection of UTI

### Biosensor-Based Technique

Every patient desires a quick, less painful diagnosis of disease, at a very initial stage, for getting an early cure. Earlier, there was used of diagnostic techniques which were labor- and time-intensive and costly too apart from the use of expensive devices. But recently, the successful use of various electrochemical biosensors was developed and used [25] for the detection of infectious diseases. Biosensor is generally used to analyses samples, to find information about their structure, constituents, and function by converting a biological response into an electrical signal [26]. The electric signal is directly proportional to the amounts of analytes, being used to obtain a measurable response. Mach et al. detailed the use of biosensors in UTI diagnosis, identification of microbes, and testing of antimicrobial susceptibility [27].

In the 1980s, there was a sensitive test for quick testing of UTI pathogens and known as *Limulus amebocytes* lysate (LAL) assay. This test was helpful for endotoxin detection [28]. Potentiometer based biosensor testing was applied for detection of *E. coli*, whenever there is a change in pH due to ammonia released by this bacterium [29]. Another sensitive biophysical technique, surface plasmon resonance (SPR), was helpful for the detection of *S. aureus* and *E. coli* [30]. There are many difficulties also while taking this biosensor technology in the detection and diagnosis of UTIs from bench to bedside.

## 4. Treatments for UTI

### 4.1. Antibiotics Prescribed in UTI

The treatment of UTI is not complicated and the disorder can be easily treated with some specific antibiotics. The irrational use of antibiotics by humans made these diseases more complicated. The treatment of UTIs can be initially limited by the intake of excess water but in case of complication (i.e., pyelonephritis or the involvement of kidney) made this disease more severe even if not treated it can cause irreversible kidney damage. It has been well documented that in most of the kidney failure cases, the presence of a UTI is the main reason after diabetes. The choice of drug or antibiotics in UTI depends on the antibiotic sensitivity testing, in Indian scenario most of the treatment being based on empirical basis without much laboratory investigation.

Another reason for ineffectiveness of antibiotics is the self-medication, without concerning to medical professional, due to economic reasons (the subjects take an inappropriate antibiotic when the case become uncontrollable, avoiding visiting the doctor). Since the first antibiotic, Penicillin, was discovered, there is a wide range of antibiotics, some of them recently discovered, part of them being effective and part of them becoming ineffective in UTI due to the body resistance development against these drugs.

The major problem associated with the administration of a suitable antibiotic, as in the majority of cases, the patient of the Indian subcontinent visits the clinician only when his condition becomes more complicated. The clinician then advised the broad-spectrum antibiotic on empirical bases and without undergoing antibiotic sensitivity testing. The antibiotic which was effective once is not as effective as it was due to the development of resistance and in place of this specific antibiotic, new or stronger/better antibiotics are prescribed (Table 2).

If we track the course of antibiotic evolution and the recent addition of antibiotics, there is no new antibiotic discovered so far, and if all antibiotics become ineffective then there is no method of chemotherapy left which can tackle the emergence or outbreak of any bacterial infection. There is an urgent need for some strategy or process which can control the common disease such as UTI, typhoid, food poisoning, and other infections. We are very fortunate to have a wealth of literature on medical practice in which there is a tradition system of medicine that has immense potential to combat most of the ailments [37].

### 4.2. Alternative Traditional Therapies for UTI Treatment

The development of resistance in uropathogens is one of the major problems associated with effective treatment in UTI. The untreated UTI may cause severe complications such as pyelonephritis or even kidney failure. In modern medicine, there is a provision of antibiotic therapy in which broad-spectrum antibiotic can be given but, these days, due to increased incidence of multidrug resistant (MDR) uropathogens, these antibiotics are ineffective and need administration of an effective antibiotic; since the development of the first antibiotic, there are only a few new antibiotics added into the list. However, this class of drugs is effective in controlling the infection, but it has also some side effects [38]. Indian patients and those abroad now know this fact, and they are focusing more on alternative medicine/traditional system of medicine. The following are few examples of alternative medicine practices in India and in many other countries.

#### 4.2.1. Homeopathic Medicine

Homeopathic medicine is one of the safest and well-known branches of alternative medicine and was developed in Germany. It is known to have no side effects, so people are taking interest in this treatment especially for small children and during pregnancy. During the gestation period, most of women encounter a UTI, and it is desirable to treat the infection, to avoid teratogenic infection. Therefore, there should be a recommendation of antibiotics, but these antibiotics may have an adverse effect on the embryo, having sometimes teratogenic effects. In such cases, there is needed of using some alternative medicine, the best alternative medicine being the homeopathic one. Nwabudike successfully treated three cases of recurrent UTI, in which the patient was given homeopathic preparations (homeopathic *Phosphorus*, *Platinum metallicum*, *Collibacillinum* and *Causticum*) [39]. De Paula Coelho et al. studied the effect of homeopathic medicine Cantharis on *E. coli*-induced cystitis and found that Cantharis modulates the uropathogenic *E. coli* (UPEC)-induced cystitis in susceptible mice [40].

Another study integrated homeopathic medicine with an antibiotic in the treatment of two cases of UTI. They used *Thuja occidentalis, Lycopodium clavatum, Sepia officinalis, Pulsatilla pratensis, sulfur, Nux vomica, Hepar Sulphur, Rhus toxicodendron, Arnica Montana, Calcarea carbonica, Tuberculinum bovinum (Kent), Natrium muriaticum, Carbo vegetabilis, Cantharis vesicatoria, Staphisagria,* and *Berberis vulgaris* along with antibiotic have good impact on the UTI [41].

The direct effect of a homeopathic drug on causal microorganism (like *E. coli*) was evaluated and it was reported that homeopathic drugs do not have any direct bactericidal and bacteriostatic effect. However, it is known they activate the immune system as well as the adhesion of bacteria on the urinary tract [42].

#### 4.2.2. Unani Medicine

Among all the known traditional system of medicine, Unani system is less popular, but it is also practiced in India and other countries. In Unani medicine system, UTI is known as Warm-e-Majra-e-Baul and the symptoms of this disorder have different terminologies such as Warm-e-Masana (cystitis), Warm-e-Kulliya (pyelonephritis), and Ufoonat (sepsis) [43]. The Unani preparation which consists of numerous ingredients (mucilage of *Althaea officinalis* L., *Sphaeranthus indicus* L., *Euphorbia hypericifolia* L., *Tribulus terrestris* L., and Sharbat Anar shireen (*Punica granatum)*, *Citrullus vulgaris* L. seeds, *Cucumis melo* L. seeds) were administered to the patients having gestational UTI. These Unani preparations were observed as being quite effective in the control of gestation UTI [44].

The Unani medicine Sat Behroza, Ral Safaid, Shora Qalmi, and Kaphoor were given to the patients in the form of powder. The administration of these drugs was given for one month and it caused significant improvement in UTI and in the urine composition [43]. Also, two Unani drugs (i.e., Safuf Mudir and Sharbat Bazuri motadil) were administered to patients who take these two drugs for 21 days; both Unani drugs had encouraging results on UTI [45]. In Unani, a system of medicine following preparation such as Habbul Aas, Mauljubn Tukhm E-Khira, Tukmm E-Kakri, Habb E Kaknaj, Sheerah Tukhm e-Khyar, annana stem water, Sheerah e-Khar e- Khasak, Sheerah Tukhm e-Khyarainin, Maaush Shaier, and Maul jubn, beneficial actions were observed in burning micturition, acidity, pyouria, and cystitis [46].

#### 4.2.3. Herbal-Based Medicine

Plants have always proved to be of therapeutic value, and the research on active substances provided by the plants has opened new options. Naturally available isothiocyanates (ITCs), which are formed after enzymatic hydrolysis of glucosinolates (that can be found in *Brassicaceae* products) may be used as alternative therapy as they are antimicrobial [47]. The roots of *Vitex negundo* and the whole plant of *Oroxylum indicum* were effective against a few selected human uropathogens [48].

In the Ayurvedic medicine system, there are certain formulations and herbs which are quite effective in controlling and treating UTI and can also help to reduce the occurrence of this disorder. Cranberry extracts have been known about for ages [49]. *Achyranthes aspera* is another herb that promotes kidney function, ensuring the proper fluid metabolism and further protecting the kidney from microbial attack. *Equisetum arvense* and *Tribulus terrestris* are mild diuretics, opening urinary channels, and acting also as mild antimicrobials [50,51]. Holy basil or *Ocimum sanctum* has been long used as a urinary antibacterial agent and helps with neutralizing the acidity in the body [50].

It has been well established that most of the bacteria causing UTIs can colonize the epithelial lining of the urinary tract and multiply there to initiate infection. The adherence ability of bacteria in these regions is due to acidic conditions and the presence of adhesin or of fimbriae. The Gram-negative bacteria or member of *Enterobacteriaceae* get access from the perineum and vagina by means of these accessories and make way to reach the urethra [52]. In Ayurveda, the diseases associated with UTI are called Vata Dosha. In Mutravahasrota roga, the urinary tract and kidneys are affected, whereas when the bladder and urethra are affected, they are known as Mutraghata Rog. The metabolic diseases where excessive urination (polyuria) occurs are called Prameha. Diuretics are the compounds that stimulate the urine flow and regulate it, helping in treating the UTI and known as Mutravirechana.

The pathogenicity of some agents is due to the presence of virulence factors (such as exotoxin, plasmid and adhesion factors (pili and fimbriae)). The major step in the pathogenicity is the adhesion to a particular cell or tissue; that adhesion is facilitated by capsule, pili, or fimbriae, and if this stem is anyhow prevented, then there are least chances of getting an infection. Some medicinal plants are well-known as having anti-adhesion activity, as follows, *Azadhirachta indica (Neem)*, *Ocimum sanctum* (Tulsi), Coriander leaf (Dhania), *Juniperus* spp., *Punica granatum* (Anaar), *Vaccinium macrocarpon* (Cranberry), *Salvia officinalis* (Salvia), *Tribulus terrestris* (Gokharu), *Terminalia chebula* (Harad), and *Cinnamomum cassia* (Dalchini) [53,54,55].

The herbs that protect the urinary bladder from uropathogens are *Equisetum arvense, Hydrangea petiolaris,* and *Zea mays* [56]. The plants like *Boerhaavia diffusa*, *Eupatorium purpureum*, *Agropyron repens*, and *Berberis vulgaris* are known to have nephroprotective activity [56]. The phenolic constituents present in the *Aronia melanocarpa*, in the form of juice, are capable of reducing the sensitivity to the pathogens [57]. Sharma et al. screened medicinal herbs against multidrug-resistant uropathogens and found that *E. coli*, *Klebsiella pneumoniae*, *Pseudomonas aeruginosa*, and *Enterococcus faecalis* can be isolated and reported that alcoholic extract of *Zingiber officinale* and *Punica granatum* inhibits the growth of *E. coli* [54]. The essential oil *Origanum majorana*, *Thymus zygis*, and *Rosmarinus officinalis* were tested against *E. coli* isolates and the highest inhibition was reported in the case of *Thymus zygis* [58].

The effectiveness of flowering plants viz. *Anogeissus acuminata*, *Azadirachta indica*, *Bauhinia variegata*, *Boerhaavia diffusa*, *Punica granatum*, *Soymida febrifuga*, *Terminalia chebula*, *Tinospora cordifolia*, and *Tribulus terrestris* were evaluated against eleven isolated uropathogens. Out of these 11 plants, three plants *A. acuminata, P. granatum,* and *S. febrifuga* were effective against all the tested uropathogens [59].

The different solvent extracts of leaves of *Clitoria ternatea* were tested against UTI caused by *Proteus mirabilis* and it was found that the maximum inhibition was seen in acetone fraction [60]. Similarly, a flavonoid extract of Algerian plant (*Marrubium vulgare* L.) possessed antibacterial potential [61]. The methanolic extract of *Hibiscus sabdariffa* was also reported to inhibit the growth of *E. coli* and *Klebsiella pneumoniae* [62]. All these important actions of the plants were highlighted in Table 3.

The main mechanism of action of most of the herbs is due to their antibacterial properties, taking into account the phytoconstituents present in the different parts of the plant (leaves, stem, flower, bark, and root). These phytoconstituents are represented by the phenols, alkaloids, terpenes, essential oils, saponins, and other secondary metabolites. The other mode of action is some plants can increase the pH of the body, a fact that influences the difficulty of bacteria accession to the urinary tract and flush out them from the body.

## 5. Bacteriophage Therapy

The world of the invisible creature was deciphered due to the invention of the microscope but the study of organisms that are smaller than the microscopic range has become possible due to the invention of the most advanced microscope (i.e., Transmission Electron Microscope and Scanning Electron Microscope). People who died because of an unknown reason now found the cause behind their disease, namely, the viruses which can easily pass through the bacteriological filters. The negative aspect of viruses was soon discovered but the positive part of discovering the viruses (i.e., the bacteriophage therapy) was known just after a long time, in the 1920s. The history of bacteriophage therapy was begun in India, when a British bacteriologist—Ernest Hankin, in 1896, observed the antibacterial properties of Ganga water against *Vibrio cholera*. The antibacterial properties are heat labile, but it can easily pass through a porcelain filter [66]. A similar finding was also observed by Frederick Twort while working on the vaccinia virus; he found that the transparent filtrate converts the bacterial culture into granules [67].

Two years later, Felix d’Herelle described a similar finding while working on a patient recovering from Shigellosis. He isolated the anti-shiga microbe from filtering the stool of the patient, and when the filtrate was added to the culture, the causal organism (i.e., *Shigella* sp.) get killed or lysed [68].

The use of bacteriophage therapy as a therapeutic agent is not practiced in India despite having the originating root of bacteriophage therapy but, in some countries, such as France and in Belgium, bacteriophage therapy is used for the treatment of patients who were not getting significant result from conventional treatments [69]. This therapy is successfully practiced and has shown great potential towards some human diseases. A study evaluated the effect of bacteriophage on patients who were suffering from UTI caused by uropathogens (*Staphylococcus aureus*, *E. coli*, *Streptococcus* spp. *Pseudomonas aeruginosa*, and *Proteus mirabilis*) and found that there were no adverse events that can be detected [70].

The adapted bacteriophage therapy might also be effective and safe for treating UTIs. The Pyo bacteriophage solution was tested against a patient with transurethral resection of the prostate, preventing UTI, and results shown the potential efficacy of this treatment [70,71]. The cocktail of commercially available bacteriophage (Pyo, Intesti, Ses, and Enko) along with 29 *E. coli* and 10 *K. pneumoniae* bacteriophages were tested against UTI isolates of *E. coli* and *Klebsiella* and it was reported the cocktail of bacteriophages showed lytic activity against these uropathogens [71].

The bacteriophage adheres to the receptor of specific uropathogens (such as coliphage on *E. coli*) and injects its genetic material inside the host. The genetic material of the host gets integrated into the genome of bacteria. The bacteria, while synthesizing their protein, start producing the bacteriophage components like the head, tail, capsid, protein coat, etc. and then finally the assembly. The production of bacteriophage in such a huge quantity causes the bacterial cell to burst and there is a release of bacteriophage after losing the bacterial cell. The most limiting factor to use the bacteriophage as therapeutic potential is the lack of many studies performed on a human [72]. Most of the research is done on mice model and, as far physiology of human and animal are considered, there is a considerable difference regarding their physiological behavior. Therefore, more animal trials are required. Another problem associated with this therapy is dose optimization. It may also possible that the immune system of the human being may resist in colonization in the gastrointestinal and urogenital organs. The basic mechanism of bacteriophage entry required attachment on the specific receptor present on the surface of bacteria, is possible that bacteria may lose their receptor and become resistant to bacteriophage [70,71]. However, regarding these issues associated with the bacteriophage, if we overcome these hurdles, bacteriophage can be the best alternative for bacterial infection.

## 6. Probiotics

The normal microbiome of a human being in the intestinal tract and genital organ is an important factor in disease prevention. It creates a competitive environment for foreign microorganisms to compete for limited space and food, and thus making it an unfavorable site for attachment and multiplication. However, if the balance of normal microflora gets disturbed by the antibiotic, weakening of the immune system and after the menopause stage, the chances of getting an infection are high. To prevent the UTI, it is necessary to restore the normal microbiome of the vagina in the female. The restoration of the vaginal microflora can be achieved through the administration of prebiotic or probiotic [73,74]. As per the U. S. Food and Drug Administration/World Health Organization (FDA/WHO) “live microorganisms that, when administered in adequate amounts, confer a health benefit on the host” is called Probiotics [75]. The concept of probiotic was given by Elie Metchnikoff, who suggested that consumption of friendly microorganisms would replace the harmful bacteria with useful bacteria. This concept of probiotic is now used by the researcher for the treatment of gastrointestinal infection and UTIs.

The comparative studies between the normal woman that do not encounter any episode of UTI vs. a woman who was suffering from recurrent UTI shown the variability in the vaginal microflora in the case of a normal woman and it was found that the normal microflora of the vagina was dominated with *Lactobacillus crispatus, Lactobacillus senii,* and *Lactobacillus iners*. However, in the case of a woman having recurrent UTIs, the presence of *Lactobacilli* species was not detected [76,77]. The presence of *Lactobacilli* spp. keeps the vaginal environment acidic due to the fermentation of lactose into lactic acid. In this acidic environment, most bacteria and yeast species are unable to proliferate or colonized and get killed. Bio-Kult Pro-Cyan is a commercially available probiotic containing two strains of *Lactobacilli* plus cranberry extracts, which was evaluated for preventing recurrent UTIs in premenopausal adult women, and it was reported that Bio-Kult Pro-Cyan was safe and effective to control UTI [78].

The high reduction in the occurrence of recurrent UTIs and the high level of colonization of *Lactobacillus crispatus* strain CTV-05 had been observed in the women who had received intravaginally the probiotic *Lactobacillus crispatus* [79]. Another study showed the intravaginal administration of *S. cerevisiae* affects the expression of virulence traits of *Candida albicans* such as aspartyl proteinases (SAPs) as well as hyphae-associated proteins (hyphal wall protein 1—HWP1 and endothelin converting enzyme—ECE1) [80]. The intraurethral administration of *Lactobacillus casei* strain Shirota against artificially induced *E. coli* in a murine model induced a significant reduction of *E. coli* and of the inflammation in the urinary tract [81]. The administration of *Lactobacillus* spp. as probiotic not only maintains the low pH of the genitourinary tract but it also produces hydrogen peroxide and inhibited the growth of *E. coli*. Probiotics also activate toll-like receptor-2 (TLR2), which produces interleukin-10 (IL-10) and myeloid differentiation factor 88 (MyD88) [82]. The use of probiotics in some countries was practiced, and it is working well [74] and regarded as safe by the Health Protection Agency Centre for Infection [83].

The probiotic bacteria inhibit or prevent the growth of uropathogens in various ways. When probiotic administrated to patients or any person, it will multiply and act as antagonistic on uropathogens by competing with the foreign pathogen for colonization and inhibiting the adherence of bacteria to the intestinal or urinary tract. It also acts by lowering the luminal pH and producing some antimicrobial compound or peptide, such as bacteriocin. Probiotic, in addition to inhibition of uropathogens, affects the mucosal cell–cell interaction by the enhancement of intestinal barrier (i.e., mucus secretion) [84].

## 7. Nanomedicine in UTI

In the last few decades, there has been burgeoning research on nanoparticles in the field of medical sciences. Nanotechnology is a newer branch of science, but it is now a well-established science worldwide. Nanotechnology is a promising field in the treatment of MDR pathogens and other medical fields. There are several reports from worldwide that show its application in the treatment of various ailments, including UTI.

The silver nanoparticles have proven to be effective against UTI, actioning versus microbes like *P. aeruginosa* and *Enterobacter* sp. [85]. The copper nanoparticles were prepared in a matrix having hydrogel (HCuNPs) and the nanoparticles displayed antibacterial activities against some selected UTI microbes; the hydrogel matrix as compared to copper nanoparticles showed less zone of inhibition against the pathogens [86]. Thus, such nanocomposites may have a role in biomedical applications. A new antibacterial agent (izohidrafural) when applied on a novel bioactive nanostructure having silica-titania sieves acted as carriers. These nanomaterials successfully inhibited the growth of a few uropathogens viz. *Klebsiella pneumoniae* and *Proteus mirabilis* [87]. The zinc oxide nanoparticles synthesized from the leaves of *Berberis aristata* have shown antibacterial activity against clinical isolates of UTI [88]. The uropathogens isolated from urine samples of the patient showed significant antibacterial activity when green synthesized ZnONPs from the leaf of *Passiflora caerulea* were tested against them [89]. The *Serratia nematodiphila*-assisted copper sulfide nanoparticles (CuS-NPs) were evaluated against uropathogens (i.e., *Escherichia coli, Staphylococcus aureus, Proteus vulgaris,* and *Klebsiella pneumoniae*) CuS-NPs, and it was found effective against the tested uropathogens [84]. These remarkable actions have been summarized in Table 4.

Divya et al. synthesized AgNPs from coral-associated bacteria [92]. The synthesized AgNPs MGL-D10 showed antimicrobial activity against UTI, causing clinical isolates (such as *Bacillus* sp., *E. coli*, *K. pneumonia*, *P. aeruginosa*, *S. aureus*, and *C. albicans*). The anti-biofilm effect of synthesized AgNPs MGL-D10 against *S. aureus* was assessed using confocal laser scanning microscopy. Catheter experiments also proved the antibiofilm and antimicrobial effect of synthesized AgNPs MGL-D10. The obtained results exhibit that the coating of synthesized AgNPs MGL-D10 on catheters effectively inhibited the growth and biofilm formation of UTI causing pathogens.

However, a hygienic lifestyle, practicing safe sex, and ingestion of adequate water can prevent the occurrence of UTIs; the route of UTI can be through the administration of contaminated catheter through the urinary tract, a fact that is essential during prostate enlargement and another ailment of the urinary tract. Another similar study was done by Yassin et al., in which they synthesized silver nanoparticle-containing self-polymerized polydopamine and it was utilized to form the coating on the surface of the catheter [5]. The developed silver nanoparticle coated catheter was reported to have antibacterial activity against uropathogens, and it was also reported to have antibiofilm and antifouling activity. The quorum sensing (QS) mediated antimicrobial agents have been suggested earlier but as per the ground, the scale is concerned the concept is not converted into product.

In most cases of the disease, there is the involvement of pathogen island or virulence factor which is controlled by the QS. If we target the QS-mediated pathogenic factors, the disease can be prevented. Qais et al. synthesized silver nanoparticles (Ag@CC-NPs) from aqueous extract of *Carum copticum*, based on the concept of QS mediated antimicrobial agents; it was found that there was significant reduction in the production of virulence factor and it was shown also antibiofilm activity by *P. aeruginosa, S. marcescens*, and *C. violaceum* [93]. These studied bacterial species are associated with catheter-associated UTIs. The coating of the catheter with this silver nanoparticle can be used for the management of UTI or other catheter induced infections.

Candida species are also related to UTIs and pose health risks. The iron oxide magnetic nanoparticles were attached to vancomycin and these conjugated nanoparticles then acted as the probe; the probes were used to trap *Staphylococcus saprophyticus* (which is a Gram-positive pathogenic bacterium) and the causal agent of UTIs in women. Moreover, such samples have been successfully used to fix *S. aureus* using a magnetic field from urine specimens [94,95]. Recently, silver nanoparticles made from *Mentha piperita* (aqueous and ethanolic extract) were used against *C. albicans*. The ethanol extract proved to be more effective as it is exposed on the surface [80].

## 8. Conclusions and Perspectives

**Figure 1:**
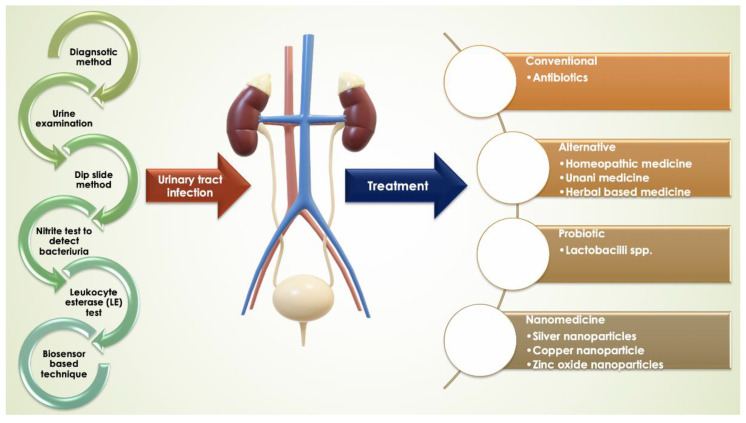
Representation of UTI, prevalence, diagnosis, and treatments.

Microbial resistance towards commonly administered antibiotics [2,96] has presented a problem to clinicians to find a permanent cure for UTIs and to lower the rate of occurrence, especially in females. There are many alternative therapies available, like home-based therapies, which are effective on case to case basis. However, other systems of medicines like Homeopathic, Unani and Ayurveda, are effective on UTIs as well. Figure 1 describes the main points and findings of this review type paper.

While finding the cure for UTIs, the focus must also be on the glycemic patients, so that the treatment being synchronous with the UTIs. These limitations have encouraged scientists to find other modern ways of treatment, with nanotechnology giving some hope, as there are reports regarding the effective use of nanoparticles against uropathogens. These findings will add further knowledge about reducing the occurrence of UTIs, thereby improving the clinical outcomes.

## Figures and Tables

**Table 1:** Colony characteristics of uropathogens on selective media.

Culture Media	Colony Characteristic/Color	Probable Bacterium
CLED	Blue green	*E. faecalis*
	Pink colony with pink halo	*E. coli*
	Golden yellow	*S. aureus*
	Orange yellow/Greenish	*Pr. mirabilis*
MacConkey Agar	Lactose fermenting pink colony	*E. coli*
	Lactose fermenting mucoid pink colony	*Klebsiella* sp.
CHROM Agar orientation (CHROM agar company, France)	Pink	*E. coli*
	Turquoise blue	*Enterococci*
	Metallic blue	*Klebsiella pneumoniae*
	Metallic blue	*Enterobacter* spp.
	Gave golden, opaque small white	*S. aureus*
CPS ID2 medium (bioMe’rieux)	Pink	*E. coli*
	Blue Green	*Enterococcus*
	Brown color	*Ptoteus mirabilis*

**Table 2:** Antibiotics prescribed for the treatment of *E. coli* UTI.

Antibiotics	Year	Ref.
Amoxicillin or bacampicillin	1990	[31]
Nitrofurantoin and trimethoprim-sulfamethoxazole (TMP/SMX)	1991–1997	[32]
Cefixime and Ofloxacin	1994	[33]
Levofloxacin	2008	[34]
Ciprofloxacin	2015	[35]
Cephalexin and Cefuroxime	2016	[12]
Cefotaxime/clavulanic acid	2020	[36]

**Table 3:** Plants active against uropathogens.

Plant	Inhibit	Fraction	Ref
*Zingiber officinale*	*E. coli*	Ethanol, *n*-Hexane	[54,63]
*Punica granatum*	*E. coli, K. pneumoniae, K. oxytoca, Proteus mirabilis, P. vulgaris, Pseudomonas aeruginosa*	Ethanol, methanol	[50,54,55,59]
*Terminalia chebula*	*K. pneumoniae, Proteus vulgaris*	Ethanol	[54,64]
*Ocimum sanctum*	*K. pneumoniae, Enterococcus faecalis*	Ethanol	[54]
*Cinnamomum cassia*	*Ps. aeruginosa*	Ethanol	[54]
*Azadirachta indica*	*E. faecalis*	Ethanol	[54]
*Thymus zygis*	*E. coli*	Essential oil	[58]
*Clitoria ternatea Marrubium vulgare* L	*Pr. Mirabilis E. coli, B. cereus, Pr. mirabilis*	Acetone Methanol	[60,61]
*Hibiscus sabdariffa*	*E. coli, K. pneumoniae*	Methanol	[62]
*Boerhavia diffusa*	*Klebsiella* sp., *Pseudomonas* sp., *Enterococcus* sp., *Escherichia coli and Proteus* sp.	Ethanol	[65]
*Vitex negundo* Linn	*E. coli, S. flexneri*	Methanol:dichloromethane (1:1)	[48]
*Oroxylum indicum*	*E. coli, S. flexneri*	Methanol:dichloromethane (1:1)	[48]

**Table 4:** Few metallic nanoparticles and their effectiveness against UTI pathogens.

Metal NP	Source	Effective Against	Ref.
ZnO-NP	*Beberis aristata*	*Escherichia coli* *,* *Staphylococcus aureus* *,* *Klebsiella pneumoniae* *,* *Bacillus subtilis* *,* *Bacillus cereus* *,* *Serratia marcescens*	[88]
ZnO-NP	*Passiflora caerulea*	*E. coli* and *Enterococcus*	[89]
CuS-NPs	*Serratia nematodiphila*	*E. coli, S. aureus, Pr vulgaris, and K. pneumoniae*	[90]
AgNP	Chemical method	*E. coli, Pseudomonas aeruginosa, Klebsiella pneumoniae, Enterobacter* sp., *Proteus morganii* and *Staphylococcus aureus*	[85]
AgNP	*Anogeissus acuminate*	*S. aureus, Enterococcus faecalis, A. baumannii, Citrobacter freundii, Enterobacter aerogenes, Escherichia coli, Klebsiella oxytoca, K. pneumoniae, Proteus mirabilis, P. vulgaris and Pseudomonas aeruginosa*	[91]
AgNP	Chemical method	*E. coli, Staphylococcus*	[7]
W-NPs	Chemical method	*E, coli, Staphylococcus*	[6]
